# Outcomes of Fasciotomy Versus Conservative Management for Chronic Exertional Compartment Syndrome: A Systematic Review and Meta-Analysis

**DOI:** 10.7759/cureus.75803

**Published:** 2024-12-16

**Authors:** Abdelfatah M Elsenosy, Ahmed Elnewishy, Eslam Hassan, Radwa A Delewar

**Affiliations:** 1 Trauma and Orthopaedics, University Hospitals Dorset, Poole, GBR; 2 Trauma and Orthopaedics, Royal Berkshire Hospital, Reading, GBR; 3 Trauma and Orthopaedics, Poole General Hospital, Poole, GBR; 4 Pharmacy, Alexandria University, Alexandria, EGY

**Keywords:** chronic exertional compartment syndrome, conservative management, fasciotomy, functional recovery, meta-analysis, pain reduction, patient satisfaction, sf-36, tegner scale

## Abstract

The aim of this systematic review and meta-analysis was to evaluate and compare the effectiveness of surgical fasciotomy and conservative management for chronic exertional compartment syndrome (CECS) concerning symptom relief, functional recovery, and patient satisfaction.

A comprehensive search of PubMed, Scopus, Google Scholar, and Cochrane Library identified studies comparing surgical fasciotomy with conservative management for CECS. Four studies met the inclusion criteria, comprising both retrospective and prospective cohort designs. The primary outcomes were pain reduction (assessed using the visual analog scale), return to activity (measured by the Tegner scale), and functional recovery (evaluated through the Short Form-36 (SF-36) quality-of-life score). Secondary outcomes included complication rates and patient satisfaction. Statistical analyses were performed to calculate standardized mean differences (SMDs) and odds ratios (ORs) with 95% confidence intervals (CIs), while heterogeneity was assessed using the I² statistic.

Surgical fasciotomy demonstrated significantly greater pain reduction compared to conservative management (SMD: -0.46, 95% CI: -0.74 to -0.17, p = 0.002), with low heterogeneity (I² = 13%). Patient satisfaction was also significantly higher in the surgical group (OR: 3.51, 95% CI: 2.19 to 5.60, p < 0.00001). However, no significant difference was observed in return-to-activity rates (OR: 3.70, 95% CI: 0.53 to 25.96, p = 0.19), with high heterogeneity (I² = 88%). Complications associated with fasciotomy included hematomas, nerve injuries, and scar-related issues, while conservative treatment showed moderate effectiveness in milder cases.

Surgical fasciotomy offers superior pain relief and higher patient satisfaction compared to conservative management for CECS. However, the evidence for return-to-activity rates remains inconsistent. Future research should focus on randomized controlled trials and standardized outcome measures to refine treatment strategies.

## Introduction and background

Chronic exertional compartment syndrome (CECS) is an activity-induced condition characterized by elevated intracompartmental pressures within fascial compartments, leading to reversible ischemic symptoms such as pain, tightness, cramping, paresthesia, and muscle weakness. These symptoms typically arise during physical activity and subside with rest. CECS predominantly affects athletic or highly active individuals and is most commonly observed in the lower legs, though it may also occur in other regions, such as the forearms or hands [1,2]. Intracompartmental pressure increases due to repetitive muscular activity within inelastic compartments, reducing tissue perfusion and causing functional impairment [3].

CECS is frequently underdiagnosed and often mistaken for other conditions, such as stress fractures or medial tibial stress syndrome. Diagnosis usually involves a combination of clinical history, imaging modalities, and direct measurement of intracompartmental pressures, confirming the condition through elevated pressures during or immediately after exercise [4].

The precise etiology of CECS remains unclear, but repetitive physical activity and mechanical stress are recognized as primary contributors. Activities involving prolonged or intense muscle use, such as running, cycling, and sports requiring repetitive gripping, are commonly associated with CECS [5,6]. Anatomical factors also play a significant role, such as tight or inelastic fascial compartments that limit muscle expansion, leading to pressure accumulation. Genetic predisposition and biomechanical abnormalities, including poor running form or overuse injuries, may exacerbate the risk [7,8]. The condition is disproportionately reported in athletes and military personnel, likely due to the high-intensity nature of their physical activities [2].

Several risk factors predispose individuals to developing CECS. Young, active individuals engaged in high-intensity or repetitive activities, such as running, cycling, and soccer, are at heightened risk. These activities often result in repetitive microtrauma or overuse injuries in the lower extremities, creating conditions conducive to CECS [9]. Biomechanical factors such as gait abnormalities, excessive foot pronation, or poor running mechanics may also contribute. Additionally, intrinsic factors like smaller, less compliant fascial compartments and genetic predispositions increase vulnerability [10]. Military personnel and athletes are commonly affected, likely due to their high levels of physical activity and repetitive load-bearing tasks [11]. Gender differences in CECS prevalence remain unclear. While some studies suggest a higher incidence in men, attributed to greater participation in strenuous physical activities, others report similar rates in women due to increasing participation in sports [12]. Factors such as smoking, higher BMI, and prolonged symptom duration before diagnosis have also been associated with poorer outcomes and greater susceptibility to CECS [13].

The pathophysiology of CECS involves elevated intracompartmental pressures during exercise, impairing vascular perfusion and neuromuscular function. Repetitive muscle contractions during exercise cause temporary muscle swelling, leading to compartmental pressures that exceed capillary perfusion pressure. This results in ischemia, pain, and neurological deficits such as paresthesia and weakness [1]. Reduced tissue compliance and insufficient fascial elasticity exacerbate the condition, as the non-compliant fascia prevents adequate expansion of the muscle compartments. Consequently, localized ischemia triggers pain and functional impairment, typically resolving at rest [14]. Emerging evidence suggests that venous outflow obstruction may also play a role, as functional compression of venous structures during exercise exacerbates compartmental pressures. This insight highlights the multifactorial etiology of CECS, combining biomechanical, vascular, and structural components [10].

Fasciotomy is a surgical procedure designed to relieve elevated pressure within a muscle compartment, commonly due to CECS. The procedure involves incising the fascia, the connective tissue surrounding the muscle compartments, to reduce pressure and restore normal blood flow and muscle function [7]. In cases of CECS, fasciotomy is considered the gold standard treatment when conservative measures fail or for patients with severe, activity-limiting symptoms [15].

Traditional open fasciotomy involves making larger incisions to release one or more compartments. While this approach ensures complete decompression, it carries a higher risk of wound complications and longer recovery times [16]. Techniques such as mini-open or ultrasound-guided fasciotomy require smaller incisions, resulting in less scarring and faster recovery. These methods have demonstrated success in reducing pain and enabling patients to return to activity more quickly [17]. Advanced techniques, including endoscopic fasciotomy, utilize minimally invasive tools to achieve decompression with reduced morbidity. Benefits include enhanced visualization, quicker rehabilitation, and fewer complications, making this approach particularly effective for athletes requiring rapid return to high-intensity sports [18].

Conservative treatment for CECS encompasses non-surgical approaches aimed at symptom relief and functional improvement. These strategies include activity modification, physical therapy, orthotic interventions, and occasionally pharmacological or injection therapies. They focus on reducing the biomechanical and physiological stresses that contribute to elevated compartment pressures during exercise, allowing patients to manage symptoms without invasive procedures [19].

Activity modification involves reducing or altering physical activities that exacerbate symptoms, such as high-impact exercises like running. Alternative activities, such as swimming or cycling, may be recommended to maintain fitness while minimizing compartmental stress. While this approach can improve symptoms for some patients, it may limit their ability to participate in desired or professional activities [20]. Physical therapy aims to enhance flexibility, strength, and neuromuscular coordination through interventions such as gait retraining, particularly for runners, and myofascial release techniques to address muscular tension. Studies have shown that targeted physical therapy can significantly reduce symptoms, allowing some patients to resume activities without surgery [21].

Emerging conservative treatments include botulinum toxin injections, which target affected muscle compartments to reduce pressure and alleviate symptoms. These interventions have demonstrated extended symptom relief, lasting several months to years [22]. Other non-surgical approaches, such as ultrasound-guided fascial fenestration and chemodenervation, show promise with long-term effectiveness and minimal adverse effects [23]. However, non-surgical treatments often yield variable outcomes, with complete symptom resolution being rare [4].

Fasciotomy remains the gold-standard treatment for CECS, particularly for patients requiring a return to high levels of activity or when conservative management fails. Satisfaction rates for fasciotomy range from 48% to 94%, with symptom recurrence reported in 10%-18% of cases [7]. Minimally invasive techniques, including endoscopic or ultrasound-guided fasciotomy, have demonstrated faster recovery times and fewer complications [18,24].

Studies comparing surgical and non-surgical approaches suggest that fasciotomy is more effective in alleviating pain and restoring activity levels. For patients with milder symptoms or lower activity demands, conservative management may be a viable option, particularly when combined with interventions like botulinum toxin injections or physical therapy [21]. Despite the promise of conservative treatments, variability in outcomes underscores the need for research to optimize management protocols, identify suitable candidates, and address key gaps in care.

The objective of this review is to evaluate and compare the effectiveness of surgical and conservative treatment approaches for CECS, with a focus on symptom relief, functional outcomes, and patient satisfaction.

## Review

Methods

Search Strategy

A comprehensive literature search was conducted in October 2024 using PubMed, Scopus, Google Scholar, and Cochrane Library to identify studies comparing surgical fasciotomy and conservative treatments for CECS. The search strategy involved a combination of Medical Subject Heading (MeSH) terms and keywords, including “chronic exertional compartment syndrome”, “fasciotomy”, “conservative treatment”, and “pain reduction”. Boolean operators (AND and OR) were employed to refine the search results, and filters were applied to include only English-language publications. To ensure the inclusion of all relevant studies, the reference lists of retrieved articles were reviewed for additional sources not identified in the initial search. The specific details of the selection process, including the inclusion and exclusion criteria applied to studies, are summarized in Table 1, which outlines the criteria used to ensure the relevance and quality of the studies included in the review.

**Table 1:** Inclusion and exclusion criteria for studies comparing fasciotomy and conservative management for chronic exertional compartment syndrome (CECS). SF-36: Short Form-36.

Criteria type	Criteria
Inclusion criteria	1. Studies were randomized controlled trials (RCTs), cohort studies, or observational studies that directly compared surgical fasciotomy and conservative treatment for CECS.
	2. Eligible studies were required to report at least one primary outcome, such as:
	- Pain intensity (measured by the visual analog scale).
	- Return to activity (e.g., using the Tegner activity scale).
	- Functional recovery (e.g., SF-36 quality-of-life score).
	3. Only studies published in English were included.
Exclusion criteria	1. Studies that did not directly compare surgical fasciotomy with conservative treatment.
	2. Case reports, editorials, opinion pieces, and conference abstracts.
	3. Studies that did not report sufficient outcome data.
	4. Studies published in a language other than English.

Measures

Primary outcomes included in this analysis were pain reduction, measured by the visual analog scale (VAS), return to sport or activity, assessed using the Tegner activity scale, and functional recovery, evaluated by the Short Form-36 (SF-36) quality-of-life score.

Secondary outcomes included complications, such as hematoma or nerve injury, patient satisfaction, assessed via Likert scales or similar measures, and return to pre-injury activity levels.

Data Extraction and Quality Assessment

Data were independently extracted by two reviewers using a standardized form to record information on study design, patient demographics, interventions, outcomes, and follow-up durations. Disagreements were resolved by consensus or by consulting a third reviewer. The quality of the included studies was assessed using the Newcastle-Ottawa Scale (NOS), which evaluates the risk of bias in selection, comparability, and outcome assessment. Each study was classified as having low, moderate, or high quality based on the NOS scores.

Statistical Analysis

Statistical analysis was conducted using RevMan version 5.4 software (Cochrane Collaboration, London, UK). For continuous outcomes, such as pain reduction and functional recovery, standardized mean differences (SMDs) with 95% confidence intervals (CIs) were calculated. For dichotomous outcomes, such as patient satisfaction and return-to-activity rates, odds ratios (ORs) with 95% CIs were computed. Heterogeneity among studies was assessed using the I² statistic, with values above 50% indicating substantial heterogeneity. A fixed-effects model was used if heterogeneity was low, and a random-effects model was applied when heterogeneity was high. Funnel plots and Egger’s test were used to assess publication bias, with statistical significance set at p < 0.05.

Results

Search and Study Selection

A structured search was conducted to identify studies comparing surgical fasciotomy and conservative treatment for CECS. The initial search yielded 80 records, with 10 duplicates removed, leaving 70 records for screening. During the title and abstract review, 50 studies were excluded as they did not meet the inclusion criteria, which focused on studies directly comparing surgical and conservative treatments for CECS.

**Figure 1:**
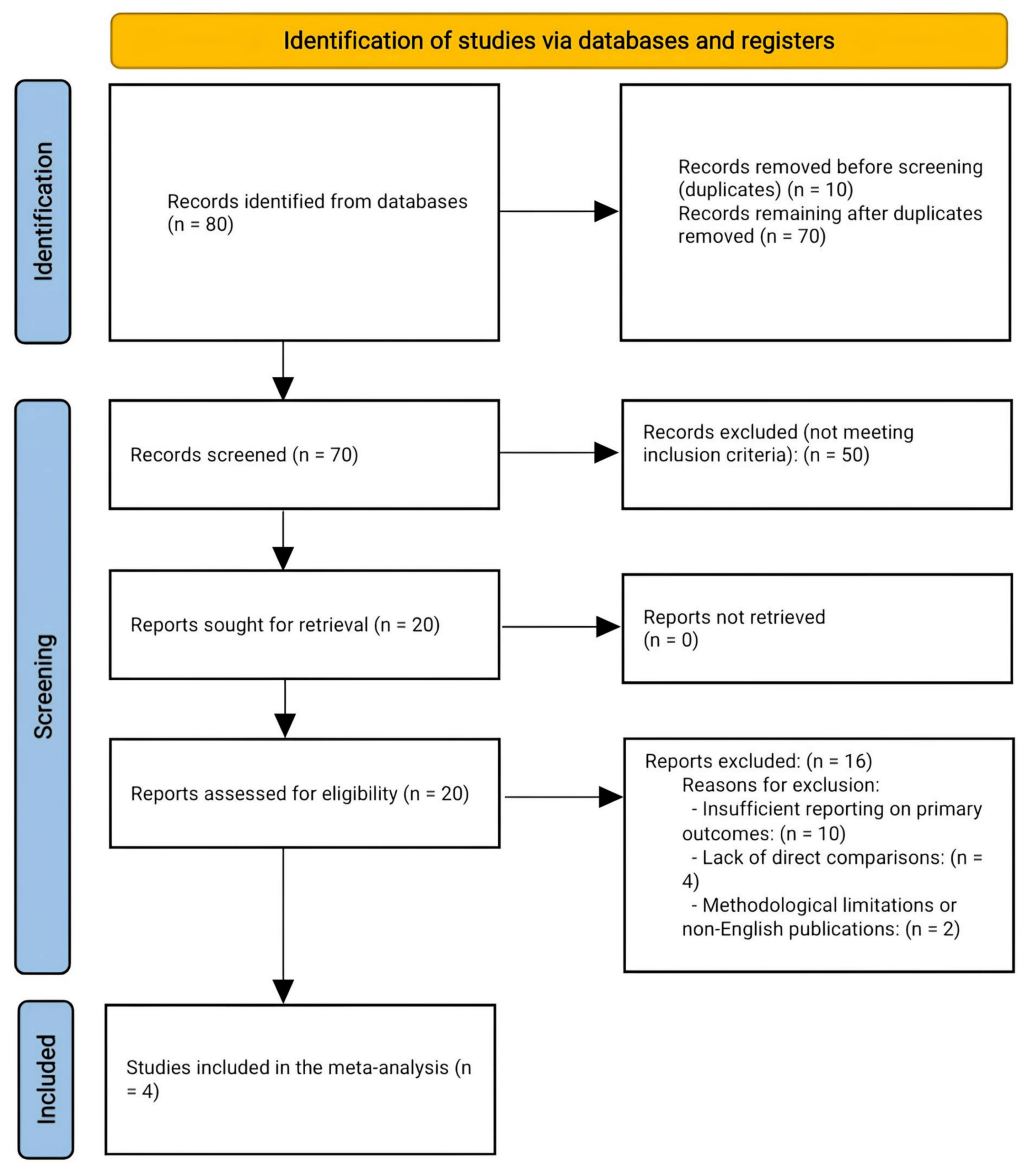
Preferred Reporting Items for Systematic Reviews and Meta-Analyses (PRISMA) flow chart showing the process of selecting studies comparing fasciotomy and conservative management for chronic exertional compartment syndrome.

Subsequently, 20 full-text articles were assessed for eligibility. After a thorough review, 16 studies were excluded due to insufficient reporting on primary outcomes (e.g., pain intensity and return to activity), methodological limitations, or non-English publications. Additionally, four studies were excluded because they lacked clear definitions of exposure or did not directly compare the two treatment modalities. Ultimately, four studies met all the inclusion criteria and were included in the final meta-analysis, as shown in Figure 1.

Study Characteristics

This meta-analysis includes four studies that compared surgical fasciotomy with conservative treatment for CECS. The studies encompassed both retrospective cohort studies and prospective observational studies, with a total sample size of 463 patients (319 in the surgical group and 144 in the conservative group). The participants were primarily active individuals aged 16-45 years, including athletes and military recruits, many of whom engaged in high-impact sports such as soccer, running, and basketball. The majority of patients had primary CECS and presented with bilateral symptoms, typically involving the anterior compartment or a combination of the anterior and posterior compartments. The interventions evaluated included surgical fasciotomy, which typically involved a 4-cm incision for anterior compartment release or combined anterior/lateral compartment release, depending on the severity of the condition. Conservative treatment involved activity modification, physical therapy, and shoe orthotics, with some patients also receiving adjunct treatments such as steroid injections or shockwave therapy. The follow-up periods across studies varied from 12 months to 5.6 years, providing a comprehensive view of both short-term and long-term outcomes for each treatment group. The primary outcomes assessed included pain intensity, measured by the VAS, return to sport or activity, evaluated using the Tegner activity scale, and overall functional recovery, assessed with the SF-36 quality-of-life score. Additionally, patient satisfaction was measured using a Likert scale, and subjective injury status was assessed via the Single Assessment Numeric Evaluation (SANE) score. Secondary outcomes included complications such as hematomas and nerve injuries, as well as the rate of return to pre-injury activity levels. The studies consistently measured subjective improvements in symptoms, particularly in terms of pain reduction and overall functional recovery, offering a robust comparison between the two treatment modalities. Table 2 summarizes the key characteristics of the included studies.

**Table 2:** Summary of study characteristics, including sample size, patient demographics, interventions, and primary outcomes for studies comparing conservative and surgical intervention for chronic exertional compartment syndrome (CECS). VAS: visual analog scale; SF-36: Short Form-36.

Category	Vogels et al. [25]	Thein et al. [26]	Packer et al. [27]	Maksymiak et al. [28]
Study design	A non-randomized cohort study comparing elective fasciotomy with conservative treatment for CECS.	A retrospective cohort study.	A cohort study evaluating the effectiveness of fasciotomy vs. conservative treatment for CECS.	A historic cohort study assessing the outcomes of surgical treatment for CECS in military and civilian patients.
Sample size	211 patients (188 surgical, 23 conservative).	43 patients (31 surgical, 12 conservative).	100 patients (73 surgical, 27 conservative).	160 patients suspected of CECS and referred for intracompartmental pressure measurement (ICPM) from 2013 to 2017.
Level of evidence	Level III (non-randomized cohort).	Level III (retrospective cohort study).	Level III (cohort study).	Level III (historic cohort study).
Patient demographics	Mean age: 30 years (surgical), 32 years (conservative). Gender: 49% male (surgical), 48% male (conservative). The majority had bilateral symptoms (80%).	Mean age: 23.8 ± 7.6 years. 72.1% underwent surgery, 27.9% were managed conservatively.	Surgical group: Mean age = 26.3 years (range 14.0-61.1 years). Predominantly young, active individuals. Conservative group: Mean age = 31.0 years (range 13.9-64.2 years).	Patients with exercise-related leg pain (ERLP); mean delay before treatment: 29.0 ± 30.3 months.
Intervention details	Surgical: Elective fasciotomy. Conservative: Physical therapy (43%), activity modification (87%), shoe orthotics (61%), steroid injections (17%), shockwave therapy (4%), dry needling (4%).	Surgical: Single 4-cm incision fasciotomy of the anterior compartment. Conservative: Rest and physical therapy for at least 6 weeks	Surgical: Fasciotomy (isolated anterior or combined anterior/lateral release). Conservative: Non-operative management with physical therapy, ice, and activity modification.	Surgical: Fasciotomy. Conservative: Activity modification and physical therapy.
Follow-up duration	12 months post-treatment.	Mean: 28.15 months (range 4.16-54.09 months)	Mean follow-up: 5.2 years (surgical), 5.6 years (conservative).	Not explicitly stated, but the study included patients referred from 2013 to 2017.
Outcome measures	Pain intensity (rest and exercise). Tightness intensity. Frequency of pain and tightness. Patient-reported satisfaction. Return to activity. Success/failure rates.	Pain: VAS score. Tegner sports activity score. Return to pre-diagnosis activity level. SF-36 quality-of-life score.	Success rate (excellent/good outcomes). Pain (analog scale). Return-to-sport rate. Patient satisfaction.	Recovery towards former performance (7-point Likert scale). Subjective injury status (Single Assessment Numeric Evaluation score). Treatment satisfaction.
Results	Baseline pain intensity: Rest = 2.5 ± 0.1 (surgical) vs. 2.0 ± 0.2 (conservative), p < 0.05; exercise = 4.2 ± 0.1 (surgical) vs. 3.8 ± 0.2 (conservative), p < 0.05. Larger pain intensity drop: Exercise: 1.6 ± 0.1 (surgical) vs. 0.9 ± 0.2 (conservative), p = 0.01. Tightness intensity drop: 1.4 ± 0.1 (surgical) vs. 0.4 ± 0.3 (conservative), p = 0.001. Frequency of tightness drop: 1.5 ± 0.1 (surgical) vs. 0.4 ± 0.4 (conservative), p = 0.001. Success rate: 42% (surgical) vs. 17% (conservative), p = 0.02. Return to previous activity: 26% (surgical) vs. 35% (conservative), p = 0.33. Failure rate: 58% (surgical) vs. 83% (conservative).	Pain improvement: The surgical group saw a greater change in pain score (ΔP = 4.27 ± 3.05) vs. the conservative group (ΔP = 1.59 ± 2.10) (p = 0.014). Return to full activity: 77.4% in the surgical group vs. 25% in the conservative group. Tegner score improvement: ΔT = 3.22 ± 3.19 (surgical) vs. ΔT = 0.09 ± 3.14 (conservative) (p = 0.009). SF-36 score: 89.2 (± 10.86) in the surgical group vs. 79.15 (± 17.50) in the conservative group (p = 0.046).	Pain reduction: Post-treatment pain scale (0-10), mean 2.2 ± 2.5 (surgical) vs. 3.7 ± 3.1 (conservative), p = 0.015. Success rate: 81% (surgical) vs. 41% (conservative) (p < 0.001). Patient satisfaction: 81% (surgical) vs. 56% (conservative) (p = 0.007). Return-to-sport: 73% (surgical) vs. 41% (conservative). Failure rate: 19% (surgical) vs. 59% (conservative). Postoperative success for isolated anterior release: 100% success vs. 31% failure for combined anterior/lateral release (p = 0.035).	Recovery: Surgically treated patients reported better recovery (2.8 ± 2.0 vs. 3.9 ± 1.7) on the Likert scale. Injury status: The surgical group had better injury status (79.3 ± 22.6 vs. 63.5 ± 27.4). Satisfaction: 75.0% in the surgical group vs. 51.4% in the conservative group.
Complications	Surgical group: Minor infection rate: 6% (managed with antibiotics); 2% temporary sensory deficits (resolved within 3 months). No reoperations for bleeding or hematoma were required.	Surgical: Hematoma (3 patients), superficial peroneal nerve sensory impairment (1 patient, temporary), long-term paresthesia (1 patient in superficial peroneal nerve area).	Surgical group: 11 complications (4 reoperations, 7 patients with neurosensory or pain complaints). No failures were reported for isolated anterior release. Hematoma: 1 case requiring reoperation. Cosmetic issues (7 female patients had complaints about scar appearance).	Not explicitly stated
Conclusions	Surgical fasciotomy significantly reduces pain, tightness, and frequency of symptoms compared to conservative treatment, with better satisfaction rates. However, the return to previous activity levels remains low in both groups.	Surgery offers advantages over conservative treatment for anterior CECS in terms of pain reduction and return to sports, but not all patients achieve full recovery.	Fasciotomy significantly improves pain relief, return to activity, and patient satisfaction compared to conservative treatment. Isolated anterior release is superior to anterior/lateral release, with a lower failure rate and better outcomes.	Surgical treatment (fasciotomy) significantly improves functional outcomes and treatment satisfaction for CECS compared to conservative treatment. Further research is needed for non-invasive diagnostic options and individualized treatment strategies.

Quality Assessment of the Included Studies

The quality of the included studies was assessed using the NOS, which evaluates study quality across key domains such as cohort selection, comparability, and outcome assessment. Each study was categorized as having low, moderate, or high quality based on their scores in these domains. The detailed NOS scores for each study are presented in Table 3.

**Table 3:** Quality assessment of included studies using the Newcastle-Ottawa Scale (NOS). * indicates a low score for the respective category. ** represents a moderate score, reflecting acceptable quality. *** denotes a high score, indicating strong quality in the respective domain.

Study	Selection	Comparability	Outcome	Total score (out of 9)
Thein et al. [26]	*	**	**	7
Vogels et al. [25]	**	**	**	6
Packer et al. [27]	***	*	***	7
Maksymiak et al. [28]	**	**	**	6

Results of meta-analysis

Pain Reduction (VAS Score)

**Figure 2:**
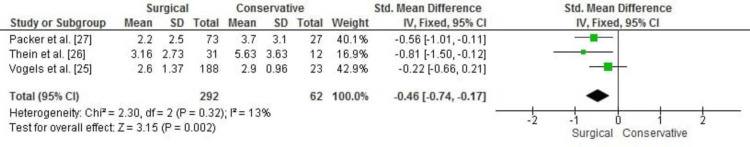
Forest plot for pain reduction (VAS score) comparing surgical fasciotomy and conservative treatment for chronic exertional compartment syndrome. VAS: visual analog scale.

The meta-analysis for pain reduction demonstrated a statistically significant difference favoring surgical fasciotomy over conservative treatment for CECS (SMD: -0.46, 95% CI: -0.74 to -0.17, p = 0.002). The heterogeneity was low (chi² = 2.30, df = 2, p = 0.32; I² = 13%), indicating consistency across the included studies. Figure 2 illustrates the forest plot for pain reduction outcomes.

Publication Bias for Pain Reduction (VAS Score)

**Figure 3:**
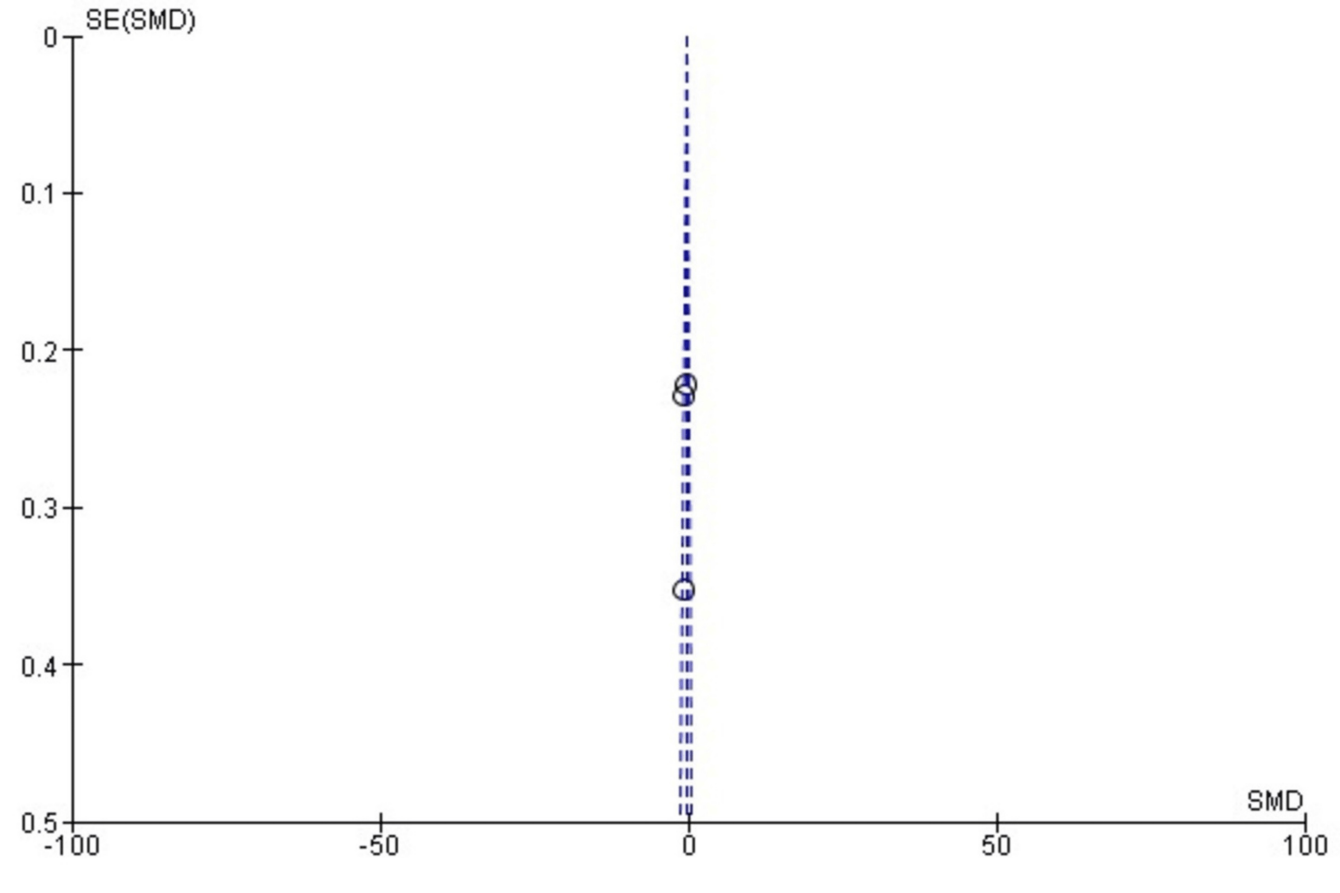
Funnel plot for pain reduction (VAS score) evaluating publication bias in studies comparing surgical fasciotomy and conservative treatment for chronic exertional compartment syndrome. The plot appears symmetrical around the standardized mean difference (SMD). This funnel plot evaluates publication bias in studies comparing surgical fasciotomy and conservative treatment for chronic exertional compartment syndrome (CECS). Data included from references [25-28]. VAS: visual analog scale.

The funnel plot for pain reduction (VAS score) appears symmetrical around the SMD, suggesting an absence of significant publication bias. This is consistent with the low heterogeneity observed in the meta-analysis (I² = 13%). Egger’s test further supports this observation, with p > 0.05. Figure 3 illustrates the funnel plot for publication bias assessment.

Treatment Satisfaction

**Figure 4:**
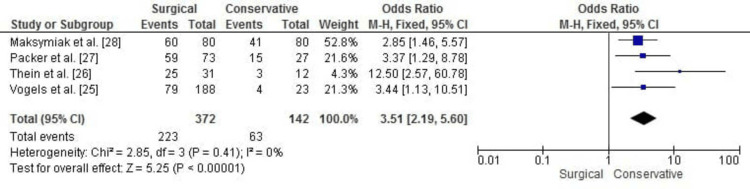
Forest plot for treatment satisfaction comparing surgical fasciotomy and conservative treatment for chronic exertional compartment syndrome (CECS) showing significantly higher odds of satisfaction.

The meta-analysis for treatment satisfaction demonstrated significantly higher odds of satisfaction for patients undergoing surgical fasciotomy compared to those receiving conservative treatment (OR: 3.51, 95% CI: 2.19 to 5.60, p < 0.00001). The results show consistency across studies, with no significant heterogeneity (chi² = 2.85, df = 3, p = 0.41; I² = 0%). Figure 4 illustrates the forest plot for treatment satisfaction outcomes.

Publication Bias for Treatment Satisfaction

**Figure 5:**
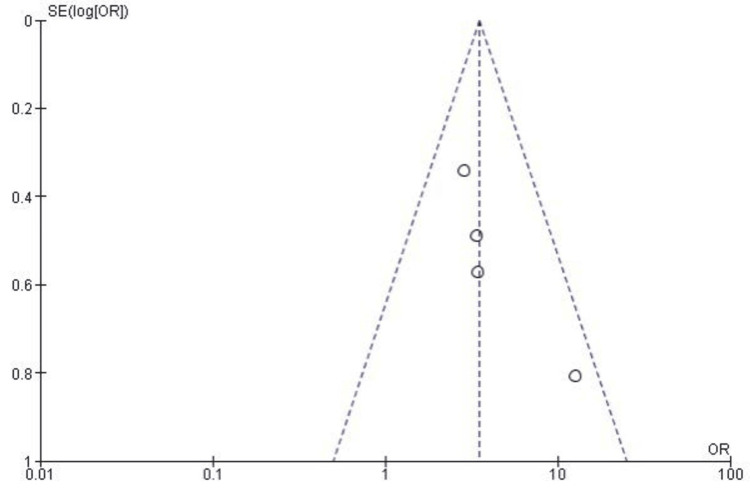
Funnel plot for treatment satisfaction in studies comparing surgical fasciotomy and conservative treatment for chronic exertional compartment syndrome (CECS) demonstrating symmetry around the odds ratio (OR). Funnel plot assessing publication bias in treatment satisfaction studies comparing surgical fasciotomy and conservative management for CECS. Data included from references [25-28].

The funnel plot for treatment satisfaction appears symmetrical around the OR, suggesting an absence of significant publication bias. This symmetry is consistent with the low heterogeneity observed in the meta-analysis (I² = 0%). Egger’s test further supports this observation, with p > 0.05. Figure 5 illustrates the funnel plot for publication bias in treatment satisfaction.

Return to Activity Level

**Figure 6:**
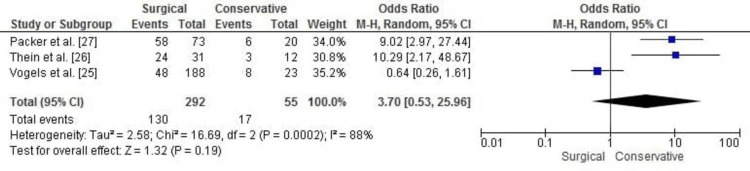
Forest plot for return to previous activity level comparing surgical fasciotomy and conservative treatment for chronic exertional compartment syndrome.

The meta-analysis for return to activity showed no statistically significant difference between surgical fasciotomy and conservative treatment for CECS (OR: 3.70, 95% CI: 0.53 to 25.96, p = 0.19). However, substantial heterogeneity was observed (chi² = 16.69, df = 2, p = 0.0002; I² = 88%), reflecting considerable variability among the studies. Figure 6 illustrates the forest plot for this outcome.

Publication Bias for Return to Activity Level

**Figure 7:**
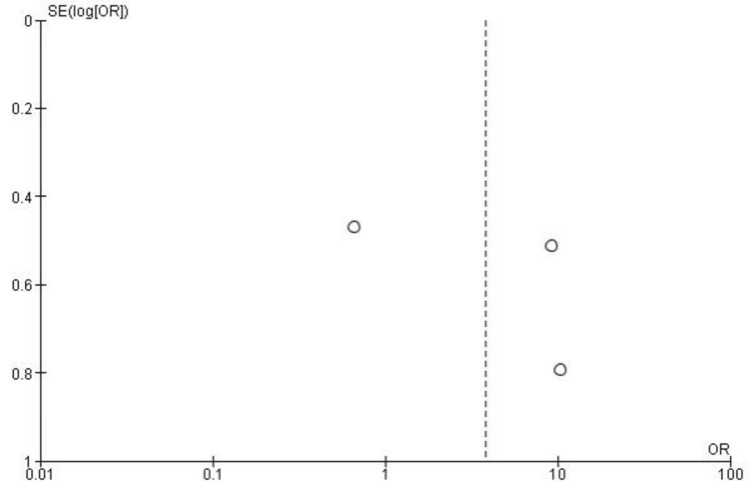
Funnel plot for return to the previous activity level in studies comparing surgical fasciotomy and conservative treatment for chronic exertional compartment syndrome (CECS) showing asymmetry around the odds ratio (OR). Funnel plot evaluating publication bias in studies analyzing return to previous activity level in CECS treatment. Data included from references [25-28].

The funnel plot for return to previous activity levels shows asymmetry around the OR, suggesting potential publication bias or heterogeneity among the included studies. This asymmetry aligns with the high heterogeneity observed in the meta-analysis (I² = 88%). However, Egger’s test indicated no significant publication bias, with p > 0.05. Figure 7 illustrates the funnel plot for this outcome.

Discussion

This systematic review and meta-analysis compared the effectiveness of surgical fasciotomy versus conservative treatment for CECS, based on outcomes such as pain relief, patient satisfaction, and return to activity. The findings from this study, supported by recent literature, offer a comprehensive understanding of the relative benefits and challenges associated with these treatment modalities. The meta-analysis found that fasciotomy significantly reduces pain intensity compared to conservative management, with SMDs favoring surgical treatment (SMD: -0.46, 95% CI: -0.74 to -0.17, p = 0.002). This result aligns with findings from Vogels et al. [25], which demonstrated a greater reduction in exercise-induced pain and tightness among surgically treated patients compared to those managed conservatively. Similarly, Ding et al. [7] suggested that fasciotomy provides substantial symptom relief, with satisfaction rates ranging from 48% to 94%. Treatment satisfaction was significantly higher for fasciotomy patients, with an OR of 3.51 (95% CI: 2.19 to 5.60, p < 0.00001). Moore et al. [29] reported high satisfaction levels in long-term follow-ups, with 72% of patients willing to undergo the procedure again. However, complications such as paresthesia and hematoma can affect overall satisfaction [30]. On the other hand, conservative management remains a feasible alternative, especially for patients with less severe symptoms or those unwilling to undergo surgery. Conservative approaches, such as activity modification, physical therapy, and gait retraining, have been shown to improve symptoms without the risks associated with surgery. Zimmermann et al. [31] found that 65% of patients managed conservatively returned to active duty without requiring surgical treatment. However, long-term adherence to conservative therapies can be challenging, with success rates declining over time. By the two-year mark, only 57% of patients maintained their activity levels without requiring surgical intervention [31]. The analysis revealed no statistically significant difference in return-to-activity rates between fasciotomy and conservative treatment. However, long-term studies suggest that fasciotomy enables higher activity levels, with 76-84% of patients returning to sports (albeit sometimes at a reduced intensity) [29,32]. Thein et al. [26] reported that 77.4% of surgically treated patients resumed their pre-diagnosis activity levels, compared to only 25% of those managed conservatively. Conversely, fasciotomy outcomes in specific populations, such as military personnel, show lower efficacy, with limited return-to-duty rates reported in studies [33]. Complications such as paresthesia and recurrence rates, ranging from 12% to 19%, highlight the risks associated with fasciotomy [30]. Alternative minimally invasive approaches, such as ultrasound-guided or endoscopic fasciotomy, have shown reduced recovery times and fewer complications, but further validation is required [24]. Conservative techniques, such as activity modification and physical therapy, offer limited success. While some patients benefit from these interventions, most eventually progress to surgical treatment [7]. In cases where surgery is not an option, emerging therapies, such as botulinum toxin injections, have shown promise in alleviating symptoms, though these are not curative [34]. Overall, conservative management lacks the immediate and significant pain relief seen with fasciotomy, making it less suitable for high-performance athletes or individuals with severe symptoms.

Limitations

Several limitations within the existing body of research should be considered. Many studies are limited by small sample sizes, retrospective designs, and a lack of standardized outcome measures. Additionally, the heterogeneity of patient populations, including variations in age, athletic activity, and symptom severity, complicates comparisons across studies. The absence of high-quality randomized controlled trials further limits the ability to draw definitive conclusions regarding the superiority of one treatment modality over the other.

## Conclusions

Fasciotomy appears to be the preferred treatment for CECS due to its superior outcomes in pain reduction and functional recovery, particularly for patients with severe symptoms. However, achieving complete functional recovery and return to pre-injury activity levels may remain challenging for some patients, regardless of the treatment approach. Future research, especially well-designed randomized controlled trials with standardized outcome measures, is essential to guide clinical decision-making and optimize treatment strategies for CECS.
